# Low-Tech Telemedicine Reduces Caregiver Burden and Improves Outcomes in Older Adults with Chronic Diseases: Results from a Prospective Study in Romania

**DOI:** 10.3390/healthcare13192442

**Published:** 2025-09-26

**Authors:** Angelica Gherman, Emil Robert Stoicescu, Codrina Mihaela Levai, Călin Marius Popoiu, Ovidiu Alin Haţegan

**Affiliations:** 1 Doctoral School Department, “Victor Babeş” University of Medicine and Pharmacy of Timisoara, 300041 Timisoara, Romania; angelica.gherman@umft.ro; 2 Research Center for Medical Communication, “Victor Babeş” University of Medicine and Pharmacy of Timisoara, Eftimie Murgu Square No. 2, 300041 Timisoara, Romania; 3 Radiology and Medical Imaging University Clinic, “Victor Babeş” University of Medicine and Pharmacy of Timisoara, Eftimie Murgu Square No. 2, 300041 Timisoara, Romania; stoicescu.emil@umft.ro; 4 Research Center for Pharmaco-Toxicological Evaluations, “Victor Babeş” University of Medicine and Pharmacy of Timisoara, Eftimie Murgu Square No. 2, 300041 Timisoara, Romania; 5 Field of Applied Engineering Sciences, Specialization Statistical Methods and Techniques in Health and Clinical Research, Faculty of Mechanics, “Polytechnic” University Timisoara, Mihai Viteazul Boulevard No. 1, 300222 Timisoara, Romania; 6 Discipline of Medical Communications, Department 2—Microscopic Morphology, “Victor Babeş” University of Medicine and Pharmacy, 300041 Timisoara, Romania; 7 Department of Pediatric Surgery, “Victor Babeş” University of Medicine and Pharmacy of Timisoara, 300041 Timisoara, Romania; 8 Discipline of Anatomy and Embriology, Medicine Faculty, Vasile Goldis Western University of Arad, Revolution Boulevard 94, 310025 Arad, Romania; hategan.ovidiu@uvvg.ro

**Keywords:** telemedicine, telehealth, caregiver burden, diabetes mellitus, stroke, dementia, chronic disease, hospital readmission, medication adherence, low-cost intervention

## Abstract

**Background**: Family caregivers of patients with chronic diseases face high levels of burden, anxiety, and burnout, which may negatively affect both their well-being and patient outcomes. Low-cost, accessible telemedicine approaches may provide practical support in resource-limited settings. **Methods**: We conducted a prospective, parallel-group controlled study in Timisoara, Romania, between April 2024 and March 2025 and included 161 caregivers of older adults with chronic diseases, allocated 1:1 to receive either structured low-cost telemedicine support (weekly phone calls and SMS reminders; *n* = 82) or usual care (*n* = 79). Data were collected at baseline, three months, and six months through structured interviews. The primary outcome was caregiver burden measured by the Zarit Burden Interview (ZBI). Secondary outcomes included caregiver anxiety, burnout, satisfaction, and patient-related outcomes such as emergency room (ER) visits, hospital readmissions, and medication adherence. Analyses were performed using ANCOVA and logistic regression, adjusting for baseline values and relevant covariates. **Results**: At three months, caregivers in the intervention group had significantly lower burden scores compared to controls (adjusted mean difference −2.9; 95% CI −4.7 to −1.1; *p* = 0.002). Reductions in anxiety (−1.4; *p* = 0.02) and burnout (−1.6; *p* = 0.01) were also observed. These effects persisted at six months, though slightly attenuated. Patient outcomes favored the intervention: ER visits were lower at six months (27.50% vs. 41.02%; aOR 0.55, 95% CI 0.30–0.99; *p* = 0.047), while medication adherence and readmissions showed consistent but non-significant improvements. No adverse effects were reported. **Conclusions**: A simple, low-tech telemedicine program reduced caregiver burden, anxiety, and burnout and decreased ER visits. Improvements in medication adherence and readmissions were observed, but did not reach statistical significance. This pragmatic intervention can easily be integrated into primary care follow-up or community-based caregiver support programs, providing an affordable and low-cost technique to promote chronic disease management and caregiver well-being, especially in resource-limited health systems.

## 1. Introduction

Chronic diseases place a considerable burden not only on patients but also on the family members who assume the role of caregivers [1,2]. Chronic diseases exert a profound impact on quality of life, affecting not only patients’ physical functioning but also their psychological well-being, social participation, and overall daily activities [3,4]. The role of family caregivers is usually undertaken by close relatives, most frequently spouses or adult children [5,6]. Despite their central role in patient care, these individuals generally had no professional training in medicine or nursing, creating a gap between the demands of caregiving and their available skills [7]. They are often responsible for monitoring symptoms, administering medications, assisting with daily activities, and ensuring timely access to medical care. Nearly half report experiencing some degree of care overload, and approximately 17% develop severe health problems, most commonly neuropsychiatric disorders [8,9]. In addition to these health consequences, family caregivers frequently encounter psychological stress, physical exhaustion, and social isolation, collectively described as caregiver burden [10]. When caregivers experience burnout or anxiety, the quality of care delivered may decline, potentially exacerbating the patient’s condition. This reciprocal relationship can place additional strain on informal caregivers [11,12].

In recent years, telemedicine has emerged as a promising tool for improving the management of chronic diseases [13,14]. As a customized medical strategy, it overcomes traditional care limits of time and distance; it also allows for virtual treatment, supervision, training, and care services and supports the allocation of medical resources [15]. Telemedicine is not limited to chronic conditions; it can also be extended to the management of inflammatory and infectious diseases, where rapid monitoring and professional support may improve outcomes and reduce complications [16,17,18]. While telemedicine often evokes high-tech platforms such as video consultations or mobile health applications, low-cost telemedicine can be implemented using far simpler tools. This is usually used as structured weekly phone calls and SMS reminders, technologies that are widely available, inexpensive, and require minimal digital literacy [19,20]. Such an approach is particularly relevant in settings where resources are limited and caregivers may not have reliable internet access or smartphones, yet still benefit from consistent contact and support. Most telemedicine interventions focus on patients rather than caregivers, and often rely on sophisticated digital platforms, applications, or remote monitoring systems that require internet access, technical skills, and financial resources [21]. This focus risks excluding populations that may benefit most, older adults, families in resource-limited settings, and socially deprived communities, where advanced telehealth infrastructure is not widely [22,23]. Telemedicine has become increasingly popular in homecare in the past few years, with implications for caregivers [24,25,26]. It could be used as a conduit for assisting caregivers of patients in palliative care, improve their overall health, and so increase the quality of care provided to patients [27].

Simple, low-cost communication methods such as telephone calls and SMS are nearly universal and require minimal technological literacy. Previous studies have demonstrated their effectiveness in supporting medication adherence, improving appointment attendance, and reducing loss to follow-up in chronic disease care [28,29]. Nevertheless, evidence regarding their impact on family caregivers remains scarce. Caregivers are rarely targeted as direct recipients of telemedicine interventions, despite being central to patient management and often the first to recognize signs of deterioration. While numerous caregiver support interventions exist, such as in-person counseling, support groups, and psychoeducational programs, these often require physical attendance, substantial time commitments, or trained personnel, which limit their scalability [30,31]. Importantly, most telemedicine initiatives have focused primarily on patients, with caregivers engaged only peripherally. Very few interventions have been designed with caregivers as the primary recipients of structured, technology-mediated support. Beyond their instrumental role, caregiver well-being has a direct and measurable impact on patient outcomes. High levels of caregiver stress and burnout have been linked to poorer adherence, delayed care-seeking, and even increased hospitalizations for patients with chronic diseases [32,33]. Supporting caregivers can improve not only their own health and resilience but also patient engagement, clinical stability, and quality of life.

Prior studies on the perception or perspective of the use of telemedicine in caring for older adults have primarily focused on the viewpoints of physicians and patients, rather than caregivers [34,35,36]. Evidence suggests that family caregivers also play a crucial role in influencing patients’ decisions to adopt and engage with telehealth technologies [37]. Physicians also recognize the involvement of caregivers in determining whether such services are utilized [34]. We hypothesized that a structured, low-tech telemedicine program designed for caregivers could reduce the burden and improve outcomes for both caregivers and patients. The present study aimed to evaluate the effectiveness of weekly telephone follow-ups supplemented by SMS reminders for family caregivers of patients with chronic diseases.

## 2. Materials and Methods

### 2.1. Study Design and Population

This study was designed as a prospective, parallel-group nonrandomized controlled study conducted between April 2024 and March 2025. The target population consisted of family caregivers of older adults with chronic diseases who were receiving follow-up care in outpatient services.

### 2.2. Inclusion and Exclusion Criteria

Caregivers were eligible for inclusion if they were the primary person responsible for daily patient care, aged 18 years or older, and had access to a mobile phone. Exclusion criteria were prior participation in structured telemedicine programs, inability to communicate via phone or messages due to hearing or literacy limitations, or refusal to provide consent.

### 2.3. Group Allocation

Caregivers were allocated in a 1:1 ratio to either the intervention or control group using simple assignment lists generated by the study team. The process did not involve allocation concealment or stratified randomization, and blinding of participants or physicians delivering the intervention was not feasible due to the nature of the program (weekly phone calls and SMS reminders). To reduce bias, interviewers performing follow-up assessments were not informed of participants’ group assignment, and data analysts worked with anonymized datasets.

### 2.4. Intervention and Outcomes

The intervention consisted of structured telemedicine support delivered through weekly phone calls and optional SMS reminders, provided over a six-month period. All phone contacts were made by a general practitioner with experience in chronic disease management, supported by a standardized protocol. The physician received training prior to the study to ensure consistency in communication style and content delivery. Each call followed a standardized checklist that covered (1) a brief assessment of the patient’s current symptoms and any changes since the last contact, (2) reinforcement of medication adherence and reminders about upcoming appointments, (3) guidance on recognizing red-flag signs that should prompt medical consultation, and (4) short supportive counseling aimed at reducing caregiver stress and providing reassurance. During the intervention, the physician was supervised by a senior clinician and had access to referral pathways for cases requiring specialized psychological or social support. Caregivers were also encouraged to raise concerns or questions during the call, allowing for individualized responses. Calls typically lasted 10–15 min. In addition, caregivers could opt to receive SMS reminders which were semi-automated but tailored to the patient’s care plan. These included reminders for medication schedules, alerts for follow-up appointments, and educational messages on recognizing warning signs. The content was drawn from a pre-developed library of messages but could be adjusted by the physician to address specific caregiver or patient needs. Messages were sent manually by the research team through a secure SMS platform to ensure accuracy and personalization. The control group received standard care, which included routine outpatient follow-up but no structured telemedicine contact. Both groups had access to usual healthcare services as required. The intervention is reported according to the TIDieR checklist (Supplementary Table S1).

The primary outcome was caregiver burden, assessed using the Zarit Burden Interview (ZBI), a validated 22-item questionnaire widely applied in caregiver research [38,39]. The ZBI was administered at baseline and again at three months and six months. In addition to the ZBI, caregiver psychological health was assessed using two validated instruments. Anxiety was measured with the Generalized Anxiety Disorder-7 (GAD-7) scale, a 7-item questionnaire scored from 0 to 21, with higher scores indicating greater anxiety symptoms [40]. Burnout was evaluated using a 3-item Emotional Exhaustion subscale adapted from the Maslach Burnout Inventory (MBI-EE), scored from 0 to 18, with higher scores reflecting higher emotional exhaustion [41]. Both instruments were administered at baseline, three months, and six months. The questionnaires are available in the Appendix A and Appendix B. They were not formally validated in Romania. For this study, they were translated and back-translated by bilingual experts.

Secondary outcomes included the occurrence of at least one emergency room (ER) visit, hospital readmission, patient medication adherence, and caregiver satisfaction with care. Medication adherence was measured through caregiver self-report, considering adherence satisfactory if at least 80% of prescribed doses were taken. Caregiver satisfaction was evaluated at three months using a Likert scale from 1 to 10, with scores ≥ 8 considered high satisfaction.

### 2.5. Data Confidentiality and Ethics Approval

All study procedures complied with the principles of the Declaration of Helsinki and applicable data protection regulations. Caregiver and patient data were de-identified and stored on secure, password-protected. SMS reminders contained only neutral information and avoided any mention of diagnoses or sensitive health details, minimizing the risk of confidentiality breaches. All participants were informed about the use of SMS and telephone communication at enrollment and provided written informed consent.

The study was conducted in accordance with the Declaration of Helsinki, and approved by the Research Ethics Committee of Victor Babes University of Medicine and Pharmacy Timisoara (approval no 77/8 January 2024).

### 2.6. Statistical Analysis

Data were collected through structured interviews conducted either in person at enrollment or via telephone at follow-up. Statistical analysis was performed using IBM SPSS Statistics, version 27.0 (IBM Corp., Armonk, NY, USA). No a priori power calculation was performed; a post hoc assessment based on observed effects was performed. Normality of continuous variables was assessed using the Shapiro–Wilk test and visual inspection of histograms. Variables approximating a normal distribution were summarized as mean ± standard deviation (SD) and compared between groups using ANCOVA adjusted for baseline values and covariates (caregiver age, sex, patient age, and disease type). Categorical variables were summarized as frequencies and percentages and compared using chi-square or Fisher’s exact tests, as appropriate Binary logistic regression was used to estimate adjusted odds ratios (aOR) and 95% confidence intervals (CI) for dichotomous outcomes. Analyses followed the intention-to-treat principle. For outcomes with >5% missing values, multiple imputation was applied using fully conditional specification with 20 imputations. The imputation model included baseline covariates and all study outcomes. Imputed datasets were analyzed separately, and estimates were pooled using Rubin’s rules. Overall, missing data were modest, averaging 3% across baseline covariates and 6–8% at follow-up outcomes Analyses of secondary outcomes were considered exploratory. No formal correction for multiple comparisons was applied, Subgroup analyses were performed stratified by patient disease type using the same analytic approach as for the main outcomes. Given the small sample sizes within each subgroup, these analyses were considered exploratory and interpreted with caution. Model fit for logistic regression was assessed using the Hosmer–Lemeshow test, Nagelkerke’s R^2^, and classification accuracy Significance was set at *p* ≤ 0.05.

## 3. Results

### 3.1. Participant Characteristics

**Figure 1:**
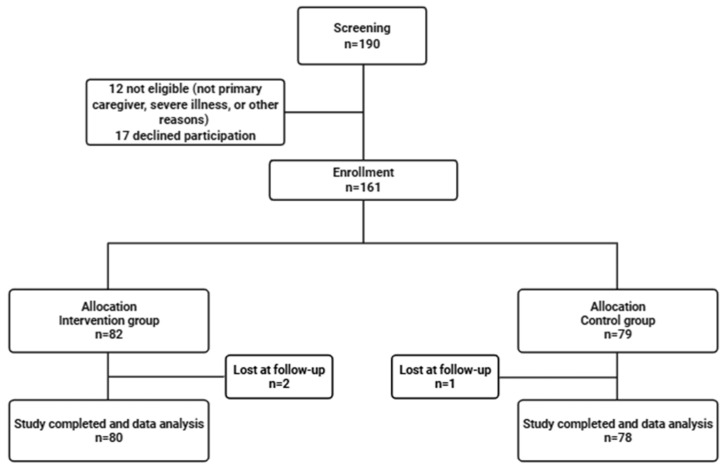
STROBE flow diagram of participant screening, enrollment, allocation, follow-up, and analysis.

A total of 190 caregivers were screened for eligibility, of whom 161 met inclusion criteria and were enrolled in the study. Eighty-two participants were allocated to the intervention group and 79 to the control group. During the follow-up period, two caregivers in the intervention group and one in the control group were lost to follow-up, resulting in 158 caregivers who completed the 6-month study. A flow diagram of participant inclusion and follow-up is shown in Figure 1.

Baseline demographic and clinical characteristics were well balanced between groups (Table 1). The mean caregiver age in the intervention group was 53.1 ± 11.8 years and 52.6 ± 12.4 in the control group, and most caregivers were female (70.73% in the intervention group and 68.35% in the control group). The majority were either spouses (40.24%/44.30%) or children (47.56%/43.03%) of the patients, while a smaller proportion were other relatives such as siblings or extended family members (12.19%/12.65%).

Patients in both groups were older adults, with a mean age of approximately 72 years (72.4 ± 8.0/71.6 ± 8.5). The distribution of chronic conditions was comparable between the intervention and control arms. In the intervention group, diabetes mellitus was present in 36.58% of patients, stroke in 34.14%, dementia in 26.82%, and other chronic diseases in 2.43%. In the control group, the proportions were similar: 35.44% diabetes mellitus, 32.91% stroke, 29.11% dementia, and 2.53% other conditions.

### 3.2. Caregiver Burden

At baseline, ZBI scores were comparable between the two study groups, so that caregivers started with similar levels of perceived burden. The mean baseline ZBI was approximately 44 in both arms, consistent with a moderate-to-high degree of caregiver stress at study entry.

At the three-month follow-up, a clear divergence between groups was observed. Caregivers who received structured telemedicine support demonstrated a significantly greater improvement compared to those in the control arm. The adjusted mean ZBI score in the intervention group decreased to 37.9, while the corresponding value in the control group was 40.5. This represented an adjusted mean difference of −2.9 points (95% CI −4.7 to −1.1; *p* = 0.002).

**Figure 2:**
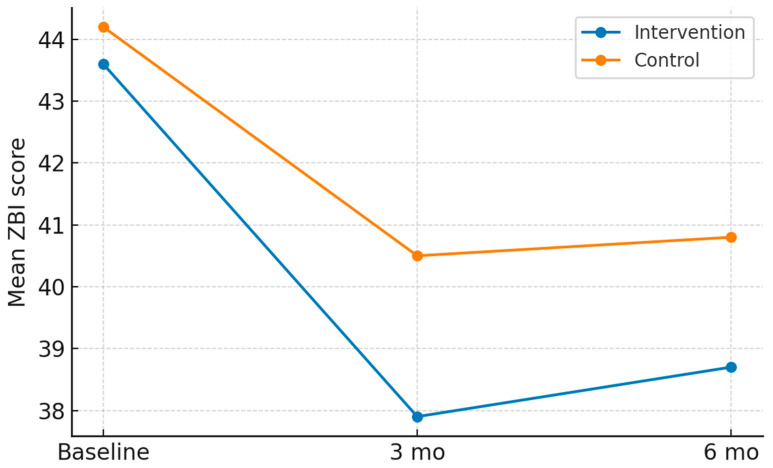
Change in Zarit Burden Interview scores at baseline, 3 months, and 6 months in the intervention and control groups.

At six months, the beneficial effect of the intervention was still evident, although the magnitude of difference between groups was slightly reduced. The adjusted mean ZBI was 38.7 in the intervention group compared to 40.8 in the control group, corresponding to a mean difference of −2.1 points (95% CI −3.9 to −0.3; *p* = 0.02). Figure 2 exhibits these trends in both groups at 3 and 6 months follow-up.

### 3.3. Anxiety and Burnout

At baseline, mean anxiety and burnout scores were similar between the two groups. Levels of anxiety were within the mild-to-moderate range across both arms, while burnout scores suggested a notable degree of emotional exhaustion even before the intervention began.

At the three-month assessment, caregivers in the intervention group reported a meaningful reduction in anxiety symptoms compared with those in the control group. The mean anxiety score decreased to 7.2 ± 3.1 in the intervention arm, whereas it remained higher in the control arm at 8.7 ± 3.0. This corresponded to an adjusted mean difference of −1.4 points (95% CI −2.6 to −0.2; *p* = 0.02). At six months, the intervention continued to show a statistically significant effect, although the magnitude of difference was slightly reduced (7.5 vs. 8.7; adjusted difference −1.1; 95% CI −2.2 to −0.1; *p* = 0.04).

A similar pattern was observed for burnout signs. At three months, mean burnout scores in the intervention group were 8.4 ± 3.5 compared with 10.0 ± 3.4 in controls, with an adjusted mean difference of −1.6 (95% CI −2.9 to −0.3; *p* = 0.01). At six months, the difference between groups persisted, though slightly attenuated, with scores of 8.6 in the intervention group and 10.0 in controls (adjusted difference −1.3; 95% CI −2.6 to −0.1; *p* = 0.04). Moreover, analyses of secondary outcomes were exploratory and no multiplicity adjustment was applied.

### 3.4. Patient Outcomes

At baseline, patient clinical profiles and risk factors were balanced between the intervention and control groups, with no significant differences in comorbidities or prior healthcare utilization.

**Figure 3:**
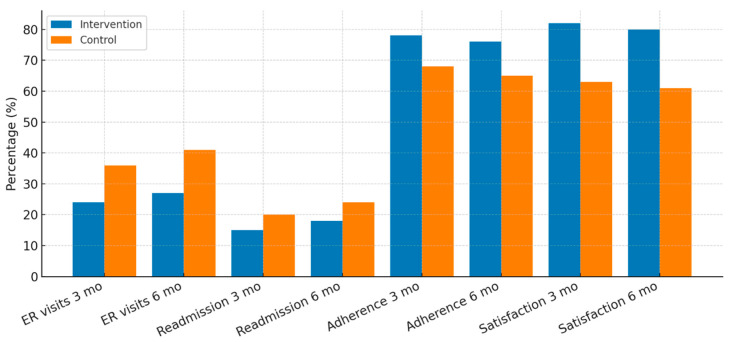
Secondary patient-related outcomes at 3 and 6 months.

At three months, 20 of 82 patients in the intervention group (24.39%) and 28 of 79 patients in the control group (35.44%) had at least one ER visit (Figure 3). Although this difference did not reach statistical significance (adjusted OR 0.58; 95% CI 0.31–1.08; *p* = 0.09), the trend favored the intervention. By six months, 22 of 80 patients in the intervention arm (27.50%) and 32 of 78 patients in the control arm (41.02%) experienced at least one ER visit. At this time point, the reduction reached statistical significance (aOR 0.55; 95% CI 0.30–0.99; *p* = 0.048).

Hospital readmissions followed a similar pattern. At three months, 12 of 82 patients in the intervention group (14.63%) required rehospitalization compared with 16 of 79 patients in the control group (20.25%). This difference was not statistically significant (aOR 0.70; 95% CI 0.32–1.52; *p* = 0.37). At six months, readmission rates were 15 of 80 patients (18.75%) in the intervention arm versus 19 of 78 (24.35%) in controls (aOR 0.72; 95% CI 0.35–1.46; *p* = 0.36).

Medication adherence also showed improvement in the intervention group. At three months, 64 of 82 patients (78.04%) achieved adherence of at least 80% of prescribed doses, compared with 54 of 79 patients (68.35%) in the control group (aOR 1.62; 95% CI 0.88–2.99; *p* = 0.12). At six months, adherence rates were 61 of 80 patients (76.25%) in the intervention arm versus 51 of 78 (65.38%) in controls (aOR 1.64; 95% CI 0.88–3.05; *p* = 0.11).

Caregiver satisfaction with the support provided was consistently higher in the intervention group across both time points. At three months, 67 of 82 caregivers (81.70%) in the intervention arm rated their satisfaction ≥ 8/10, compared with 50 of 79 caregivers (63.29%) in the control group (aOR 2.60; 95% CI 1.32–5.12; *p* = 0.006). High satisfaction was sustained at six months, with 64 of 80 caregivers (80.00%) reporting scores ≥ 8/10 compared with 48 of 78 (61.53%) in the control group (aOR 2.41; 95% CI 1.23–4.72; *p* = 0.01). All logistic regression models demonstrated acceptable fit (Hosmer–Lemeshow test, all *p* > 0.20). The explanatory power was modest, with Nagelkerke’s R^2^ ranging from 6% to 12%. Overall classification accuracy ranged between 69% and 78% across outcomes. Table 2 presents primary and secondary outcomes at 3 and 6 months.

### 3.5. Subgroup Analyses

We conducted exploratory subgroup analyses by patient disease type (diabetes, stroke, dementia). At 6 months, adjusted reductions in caregiver burden (ZBI score) were −2.8 (95% CI −4.9 to −0.7) for caregivers of patients with diabetes, −3.1 (95% CI −5.3 to −0.9) for those caring for stroke patients, and −2.4 (95% CI −5.2 to 0.4) for caregivers of dementia patients. Although the effect estimates varied slightly, the overall interaction test was not statistically significant (*p* = 0.42). For ER visits at 6 months, the adjusted OR for intervention versus control was 0.55 (95% CI 0.25–1.20) among caregivers of diabetes patients, 0.50 (95% CI 0.21–1.18) among caregivers of stroke patients, and 0.62 (95% CI 0.23–1.70) among caregivers of dementia patients. Again, the disease type × intervention interaction was not significant (*p* = 0.56). Due to very small numbers, patients with other chronic conditions (*n* = 4) were not analyzed separately. Results should be interpreted as exploratory given the limited sample sizes.

## 4. Discussion

This study evaluated the impact of a low-tech telemedicine intervention on caregiver burden, caregiver psychological well-being, and patient outcomes in the context of chronic disease management. Telehealth provides significant advantages, including improved access to medical services, higher-quality patient–provider interactions, and reductions in both costs and time commitments [42]. During the COVID-19 pandemic, its adoption accelerated rapidly, becoming an integral component of healthcare delivery across multiple specialties [43]. Our results demonstrate that this pragmatic approach significantly reduced caregiver burden, anxiety and burnout and also reduced ER visits. Improvements in medication adherence and readmissions were observed, but these did not reach statistical significance.

Caregivers receiving structured telemedicine support experienced a greater reduction in perceived burden. Which, although modest in magnitude, is likely to be clinically meaningful, as prior research indicates that even small improvements translate into better caregiver well-being and resilience [44,45,46]. Although some studies have included the ZBI as an outcome measure, none have reported a minimal clinically important difference for this scale [47,48]. Our findings also extend beyond burden, even though the reductions in anxiety and burnout observed in our study are modest. Meta-analyses in caregiver interventions often report small effect sizes in this domain. Hansen et al. found a pooled effect size of ~−0.32 (95% CI −0.53 to −0.11) for psychological distress across various caregiver populations [49]. Wei et al. reported a standardized mean difference of −0.32 (95% CI −0.50 to −0.14) for anxiety among dementia caregivers [50].

The most clinically relevant finding for patient outcomes was the significant reduction in ER visits at six months. Avoiding unnecessary ER utilization not only reduces costs and pressure on acute care services but also spares patients and caregivers from the stress of unplanned hospital visits. Even a modest relative reduction, as observed in our study, has important implications for healthcare planning, particularly in resource-limited systems where ER overcrowding is common [51,52]. Integrating caregiver-focused telemedicine support into chronic disease management pathways could therefore contribute meaningfully to reducing acute care demand. Caregivers of patients with dementia often face high emotional and behavioral challenges [53], while those caring for stroke survivors frequently manage physical disability and rehabilitation needs [54]. In contrast, caregivers of patients with diabetes may deal more with routine monitoring and medication adherence [55].

Our findings are consistent with prior studies demonstrating the benefits of caregiver-focused interventions in chronic disease management. Previous studies of more complex digital platforms have shown improvements in adherence and caregiver coping, but these approaches often require internet access [56] or advanced applications and devices [57,58], limiting their applicability in resource-constrained settings. More recently, artificial intelligence (AI) has also been explored in telemedicine [59]. In contrast, our intervention relied only on phone and messaging technologies that are widely available. Few prior studies have specifically targeted caregiver outcomes using such low-tech approaches, making this work a valuable contribution to the literature on accessible telemedicine. Zhu et al. concluded in their systematic review that telehealth interventions significantly reduced caregiver burden, depression, and stress among caregivers of people with dementia, while effects on anxiety remained inconclusive. Individualized interventions outperformed standardized techniques, and both Internet and telephone delivery were useful [60]. However, other studies found benefits mainly in improving caregivers’ self-efficacy, with no statistically significant differences observed for burden, stress, depression, or quality of life [61]. Another systematic review regarding informal caregivers of stroke survivors reported variable results. Depression was one of the most frequently investigated outcomes, yet many studies failed to demonstrate significant improvements, highlighting variability in results [62]. Our results align with evidence from caregiver support groups and psychosocial interventions. Yang et al. concluded in a systematic review that while telemedicine effectively alleviates caregiver burden and anxiety, its impact on depression and quality of life is less certain, not statistically significant, but with potential clinical benefit [63]. Caregivers in the study of Guo et al. emphasized that telemedicine could ease the burden of home care by providing timely professional support in unexpected situations and improving access to healthcare services despite distance or pandemic restrictions. They also expressed strong needs for symptom management guidance, emotional support, daily life care advice, and easy-to-use telemedicine systems with real-time responses and personalized reminder [64].

Many caregivers rely on mobile phones with limited functionality, which restricts the use of telehealth services such as video consultations or online health record access [65,66]. Barriers also arise from limited awareness of telehealth and reluctance to share health information online due to privacy concerns [67]. Insufficient dissemination of knowledge by healthcare professionals further reduces adoption [68]. To improve accessibility, telehealth systems should be simplified, for example, through larger fonts, visual aids, and streamlined interfaces [69], while providers strengthen trust and therapeutic relationships to encourage caregiver engagement [70].

Beyond telemedicine, a wide range of caregiver support strategies have been studied, including psychoeducational counseling [71], structured support groups [31], and nurse-led home visits [72]. These programs often reduce caregiver stress and improve coping skills, but they require significant healthcare resources, trained personnel, and the caregiver’s ability to travel or commit time to regular sessions. While such models may be effective in well-resourced settings, they are less feasible in socially deprived or rural communities where healthcare access is already limited. Similarly, digital health platforms and caregiver-specific mobile applications have shown promise in enhancing engagement and self-efficacy, but they presuppose digital literacy, reliable internet access, and sometimes additional financial costs [73].

This study was conducted in Romania, where family caregiving plays a central role and where primary care is relatively accessible. These cultural and structural factors may have influenced both the effectiveness and acceptability of the intervention. While weekly calls and SMS messages are low-cost and technically feasible in many settings, the degree of caregiver engagement and impact may vary depending on local caregiving norms, health system infrastructure, and cultural expectations of family support.

A major strength of this study is its pragmatic design, focusing on simple tools that can be implemented in diverse healthcare systems without significant financial investment. The use of internationally recognized, validated instruments (ZBI, GAD-7, MBI-EE) enhances comparability with prior research. The inclusion of both caregiver-focused and patient-focused outcomes provides a comprehensive picture of the intervention’s impact. Furthermore, the six-month follow-up allowed us to demonstrate that benefits were not transient but sustained over time, albeit with some attenuation.

Another important strength of this intervention is its scalability. Because telephone calls and SMS reminders are nearly universal, the program can be implemented in diverse healthcare systems without requiring new infrastructure or costly digital platforms. This is particularly relevant in low- and middle-income countries, where internet access, digital literacy, and financial resources for advanced telehealth solutions are often limited. Integrating structured, low-tech telemedicine into existing primary care or community health worker programs could offer a cost-effective strategy to reduce caregiver burden while improving patient outcomes. Policymakers may also consider embedding such interventions into chronic disease management guidelines, where they could complement in-person visits, reduce unnecessary emergency department use, and relieve pressure on overburdened healthcare system.

Several limitations must also be acknowledged. The study was conducted in a single center with a relatively modest sample size, which may have limited statistical power for some secondary outcomes. Self-reported measures of adherence and satisfaction are also subject to bias. The reduction in emergency room visits at six months was statistically significant and supports the hypothesis that proactive caregiver support can help prevent avoidable acute care use. However, the improvements in medication adherence and hospital readmission, while consistently favoring the intervention group, did not reach statistical significance. These findings should therefore be interpreted with caution. Nonetheless, the direction of the effect suggests potential benefit, which should be confirmed in larger studies with objective adherence measures.

The post hoc assessment based on observed effects indicated adequate power for the 3-month ZBI outcome, but insufficient power for smaller 6-month ZBI differences and several secondary outcomes. Future multicenter studies with larger samples (≥140 participants per arm to secure the smaller ZBI effects, and ≥200–270 per arm if ER reduction or adherence are primary endpoints will be necessary to confirm and extend these results.

Finally, although the intervention showed benefits at six months, the attenuation of effects suggests that ongoing reinforcement may be necessary to maintain long-term gains.

The findings of this study have important implications for clinical practice and health policy. By targeting caregivers directly, low-tech telemedicine can provide an accessible means of reducing burden and psychological distress, thereby supporting both caregiver well-being and patient outcomes. Integration of structured phone-based follow-up into routine chronic disease programs could be a cost-effective strategy, particularly in settings where advanced telehealth infrastructure is not feasible.

Maintaining long-term impact will likely require ongoing reinforcement. Strategies such as extending the frequency or duration of follow-up calls, transitioning from weekly to monthly check-ins after the initial intensive phase, or integrating caregiver support into routine primary care visits could help preserve gains. Future research should focus on larger, multi-center trials to confirm these findings, explore cost-effectiveness, and examine longer-term outcomes beyond six months. Combining low-tech support with community resources or periodic in-person reinforcement could also help sustain improvements over time.

## 5. Conclusions

This study shows that low-tech telemedicine given via structured phone conversations and simple messages has significant benefits for caregivers and patients managing chronic conditions. This technique provides a scalable and equitable strategy for strengthening chronic disease management, particularly in underserved groups, by reducing caregiver burden, anxiety, and burnout and decreasing ER visits. While improvements in adherence and readmission were observed, these did not reach statistical significance and should be considered exploratory. To translate these findings into practice, telephone follow-up programs could be integrated into primary care and community-based caregiver support services, offering a feasible, low-cost model for supporting families and reducing pressure on healthcare systems, especially in resource-limited settings.

## Figures and Tables

**Table 1:** Baseline Characteristics of Caregivers and Patients.

Characteristic	Intervention (*n* = 82)	Control (*n* = 79)	*p*-Value
Caregiver age, mean ± SD	53.1 ± 11.8	52.6 ± 12.4	0.79
Female caregiver, *n* (%)	58 (70.73%)	54 (68.35%)	0.88
Relationship to patient			
– Spouse	33 (40.24%)	35 (44.30%)	0.72
– Child	39 (47.56%)	34 (43.03%)	0.68
– Other	10 (12.19%)	10 (12.65%)	1
Patient age, mean ± SD	72.4 ± 8.0	71.6 ± 8.5	0.54
Primary chronic disease			
– Diabetes mellitus	30 (36.58%)	28 (35.44%)	0.81
– Stroke	28 (34.14%)	26 (32.91%)	1
– Dementia	22 (26.82%)	23 (29.11%)	0.88
– Others Baseline disease severity Charlson Comorbidity Index (mean ± SD) ≥1 hospitalization in prior 12 months, *n* (%)	2 (2.43%) 4.1 ± 1.3 29 (35.36%)	2 (2.53%) 4.0 ± 1.4 28 (35.44%)	1 0.64 0.99

Abbreviations: *n*—number; SD—standard deviation.

**Table 2:** Primary and Secondary Outcomes at 3 and 6 Months.

Outcome	Intervention 3 mo (*n* = 82)	Control 3 mo (*n* = 79)	Adjusted Effect (95% CI)	*p*-Value	Intervention 3 mo (*n* = 80)	Control 3 mo (*n* = 78)	Adjusted Effect (95% CI)	*p*-Value
ZBI, mean ± SD	37.9 ± 6.1	40.5 ± 6.3	−2.9 (−4.7 to −1.1)	0.002	38.7 ± 6.0	40.8 ± 6.2	−2.1 (−3.9 to −0.3)	0.02
Anxiety score, mean ± SD	7.2 ± 3.1	8.7 ± 3.0	−1.4 (−2.6 to −0.2)	0.02	7.5 ± 3.2	8.7 ± 3.1	−1.1 (−2.2 to −0.1)	0.04
Burnout score, mean ± SD	8.4 ± 3.5	10.0 ± 3.4	−1.6 (−2.9 to −0.3)	0.01	8.6 ± 3.4	10.0 ± 3.6	−1.3 (−2.6 to −0.1)	0.04
≥1 ER visit, *n* (%)	20 (24.39%)	28 (35.44%)	0.58 (0.31–1.08)	0.09	22 (27.50%)	32 (41.02%)	0.55 (0.30–0.99)	0.047
Readmission, *n* (%)	12 (14.63%)	16 (20.25%)	0.70 (0.32–1.52)	0.37	15 (18.75%)	19 (24.35%)	0.72 (0.35–1.46)	0.29
Medication adherence ≥ 80%, *n* (%)	64 (78.04%)	54 (68.35%)	1.62 (0.88–2.99)	0.12	61 (75.25%)	51 (65.38%)	1.64 (0.88–3.05)	0.10
Satisfaction ≥ 8/10, *n* (%)	67 (81.70%)	50 (63.29%)	2.60 (1.32–5.12)	0.006	64 (80.00%)	48 (61.53%)	2.41 (1.23–4.72)	0.01

Abbreviations: CI—confidence interval; ER—emergency room; mo—months ZBI—Zarit burden interview.

## Data Availability

The data presented in this study are available on request from the corresponding author due to privacy and ethical restrictions.

## Supplementary Materials

The following supporting information can be downloaded at: https://www.mdpi.com/article/10.3390/healthcare13192442/s1, Table S1. TIDieR checklist for reporting the intervention.

## Abbreviations

The following abbreviations are used in this manuscript:

AI	Artificial Intelligence
aOR	Adjusted Odds Ratio
CI	Confidence Interval
ER	Emergency Room
GAD-7	Generalized Anxiety Disorder-7
MBI-EE	Maslach Burnout Inventory Emotional Exhaustion Subscale
SD	Standard Deviation
SMS	Short Message Service
ZBI	Zarit Burden Interview

## Appendix A

**Table A1:** Generalized Anxiety Disorder-7 Scale.

Item (Past 2 Weeks, How Often Have You Been Bothered By…)	Response Options (0–3)
1. Feeling nervous, anxious, or on edge	0 = Not at all; 1 = Several days; 2 = More than half the days; 3 = Nearly every day
2. Not being able to stop or control worrying	0 = Not at all; 1 = Several days; 2 = More than half the days; 3 = Nearly every day
3. Worrying too much about different things	0 = Not at all; 1 = Several days; 2 = More than half the days; 3 = Nearly every day
4. Trouble relaxing	0 = Not at all; 1 = Several days; 2 = More than half the days; 3 = Nearly every day
5. Being so restless that it is hard to sit still	0 = Not at all; 1 = Several days; 2 = More than half the days; 3 = Nearly every day
6. Becoming easily annoyed or irritable	0 = Not at all; 1 = Several days; 2 = More than half the days; 3 = Nearly every day
7. Feeling afraid, as if something awful might happen	0 = Not at all; 1 = Several days; 2 = More than half the days; 3 = Nearly every day

## Appendix B

**Table A2:** Maslach Burnout Inventory (3-item short form).

Item (How Often Do You Feel…)	Response Options (0–6)
1. I feel emotionally drained from my responsibilities.	0 = Never → 6 = Every day
2. I feel used up at the end of the day.	0 = Never → 6 = Every day
3. I feel fatigued when I get up in the morning and have to face another day.	0 = Never → 6 = Every day
