# Supplementary material for: Low-Tech Telemedicine Reduces Caregiver Burden and Improves Outcomes in Older Adults with Chronic Diseases: Results from a Prospective Study in Romania
Source: Healthcare (Basel). 2025 Sep 26;13(19):2442. doi: 10.3390/healthcare13192442 (PMC12524261; doi:10.3390/healthcare13192442)
Supplement: Supplementary file 1 [file healthcare-13-02442-s001.zip › healthcare-3861246-supplementary.pdf]

Supplementary Table S1. TIDieR checklist for reporting the intervention.

| Item | Description for this study |
|---|---|
| **1. Brief name** | Structured telemedicine support program for family caregivers of patients with chronic diseases. |
| **2. Why (rationale)** | To reduce caregiver burden, anxiety, and burnout by providing structured weekly support, education, and reminders, thereby improving patient outcomes (adherence, reduced acute care use). |
| **3. What (materials)** | (a) A standardized checklist for weekly phone calls (covering symptom monitoring, medication adherence, red-flag signs, caregiver stress). (b) A pre-developed SMS message library including reminders for medications, follow-up visits, and red-flag warnings. (c) A secure phone line and SMS platform managed by the study team. |
| **4. What (procedures)** | Weekly telephone calls with caregivers, including the following: (1) assessment of patient status and recent changes; (2) reminders for medication adherence and upcoming visits; (3) guidance on recognizing red-flag signs; (4) supportive counseling for caregiver stress. Caregivers could raise additional questions. Optional SMS reminders were sent 2–3 times per week based on the patient's care plan. |
| **5. Who provided** | Calls were delivered by a general practitioner with training in chronic disease management and supportive communication. SMS reminders were coordinated by the study team under physician supervision. |
| **6. How (mode of delivery)** | One-to-one telephone calls; SMS text messages sent to caregivers' mobile phones. |
| **7. Where** | Calls were placed from the university hospital telemedicine unit. Caregivers received calls and SMS at home or in their usual setting. |
| **8. When and how much** | Weekly telephone calls (~10–15 minutes each) for 6 months. SMS reminders 2–3 times per week, tailored to patient needs. |
| **9. Tailoring** | SMS content was semi-standardized but tailored to each patient's care plan ( diabetes medication timing vs. stroke rehabilitation follow-ups). Caregivers could opt in or out of SMS support. |

| Item | Description for this study |
|---|---|
| **10. Modifications** | No major modifications to the intervention protocol occurred during the study. |
| **11. How well (planned fidelity)** | Use of a standardized checklist to structure calls and ensure consistent content across caregivers. |
| **12. How well (actual fidelity)** | Call logs tracked adherence to the weekly schedule; >90% of planned calls were delivered. SMS reminders were sent as planned in >85% of cases. |
